# TCP Transcription Factors in Plant Reproductive Development: Juggling Multiple Roles

**DOI:** 10.3390/biom13050750

**Published:** 2023-04-26

**Authors:** Ivana L. Viola, Daniel H. Gonzalez

**Affiliations:** Instituto de Agrobiotecnología del Litoral (CONICET-UNL), Cátedra de Biología Celular y Molecular, Facultad de Bioquímica y Ciencias Biológicas, Universidad Nacional del Litoral, Santa Fe 3000, Argentina

**Keywords:** flower development, flower symmetry, flowering time, inflorescence architecture, reproductive strategy, TCP domain, zygomorphy

## Abstract

TEOSINTE BRANCHED1/CYCLOIDEA/PROLIFERATING CELL FACTOR (TCP) transcription factors (TFs) are plant-specific transcriptional regulators exerting multiple functions in plant growth and development. Ever since one of the founding members of the family was described, encoded by the *CYCLOIDEA* (*CYC*) gene from *Antirrhinum majus* and involved in the regulation of floral symmetry, the role of these TFs in reproductive development was established. Subsequent studies indicated that members of the CYC clade of TCP TFs were important for the evolutionary diversification of flower form in a multitude of species. In addition, more detailed studies of the function of TCPs from other clades revealed roles in different processes related to plant reproductive development, such as the regulation of flowering time, the growth of the inflorescence stem, and the correct growth and development of flower organs. In this review, we summarize the different roles of members of the TCP family during plant reproductive development as well as the molecular networks involved in their action.

## 1. Introduction

**Figure 1:**
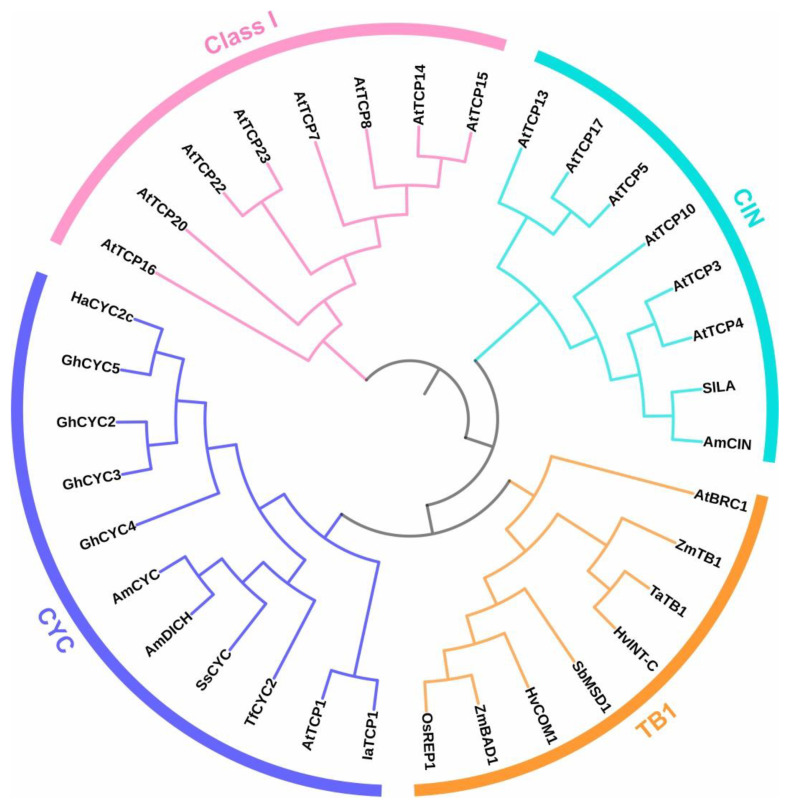
A schematic tree showing the relationships among members of the TCP family mentioned in this article. Class I TCPs are shown in pink, while members of class II are divided into three groups: CIN (cyan), TB1 (orange), and CYC (purple). Note the presence of two different groups of CIN TCPs: those that are targets and those that are not targets of miR319 (AtTCP5, AtTCP13, and AtTCP17 belong to this last group). In addition, note that CYC and TB1 groups actually belong to the same clade, named CYC/TB1 or ECE, which is separated here as a result of the specific members that were selected. In fact, the group named CYC contains essentially members of the CYC2 clade that evolved in dicots and is related to floral symmetry (see Section 3 for details), while the group named TB1 contains members of the CYC/TB1 groups 18, 19, and 20 that evolved in grasses and are related to inflorescence architecture, in addition to shoot branching, together with *A. thaliana* BRC1 (see Section 6 for details). Species abbreviations are Am, *Antirrhinum majus*; At, *Arabidopsis thaliana*; Gh, *Gerbera hybrida*; Ha, *Helianthus annuus*; Hv, *Hordeum vulgare*; Ia, *Iberis amara*; Os, *Oryza sativa*; Sb, *Sorghum bicolor*; Sl, *Solanum lycopersicum*; Ss, *Sinningia speciosa*; Ta, *Triticum aestivum*; Tf, *Torenia fournieri*; Zm, *Zea mays*. Full-length protein sequences were aligned with Clustal Omega (https://www.ebi.ac.uk/Tools/msa/clustalo/, accessed on 18 November 2022) and used to construct the tree using the Neighbor-Joining method. Bootstrap analysis was not performed. The tree was displayed using iTOL (https://itol.embl.de/itol.cgi, accessed on 19 April 2023). The node of class I TCPs was used as the root for better visualization. For further details about the phylogeny of TCP proteins, the reader is referred to recently published papers [5,8].

TCP transcription factors constitute a plant-specific protein family which is conserved in the plant kingdom and was first described in the late 1990s. TCP is an acronym of the name of the founding genes isolated from three species: *TB1* (*TEOSINTE BRANCHED 1*) from maize (*Zea mays*), *CYCLOIDEA* (*CYC*) from snapdragon (*Antirrhinum majus*), and *PROLIFERATING CELL FACTOR 1* and *2* (*PCF1* and *2*) from rice (*Oryza sativa*) [1]. TCP proteins (TCPs) contain a highly conserved domain of about 60 amino acids, located towards the amino terminus of the protein, called TCP, which has the ability to bind specific DNA sequences, establish protein–protein interactions, and is involved in protein nuclear localization [1,2]. According to sequence differences both inside and outside the TCP domain, the family is divided into two subfamilies or classes: I and II. A four-amino-acid deletion within the TCP domain of class I TCPs is the main distinction between both classes. Class II TCP members are further divided into two groups or clades: the CIN group, ubiquitous in the plant kingdom, and the CYC/TB1 angiosperm-specific group [3] (Figure 1). The CIN clade is further divided into two groups based on the existence of a microRNA-binding site located outside the TCP domain, closer to the 3′ part of the coding region. Outside the TCP domain, various members of class II (mostly CYC/TB1 proteins) also present a conserved 18–20 amino acid arginine-rich motif (R-domain) that forms an α-helix structure that coils similarly to leucine zippers, but of unknown functions. In addition, most CYC/TB1 members have a conserved motif (ECE) between the TCP- and R-domains [4]. Within class II, the CIN clade has been proposed to be more ancestral than the CYC/TB1 clade, which is absent in non-vascular plants [5]. Nonetheless, since lower plants carry both classes of TCPs, the actual ancestor of the TCP family remains unknown [3]. Over the years, TCP family members have been identified in various species but mainly characterized in Arabidopsis. The Arabidopsis genome encodes 13 class I and 11 class II TCPs, of which 3 belong to the CYC/TB1 clade [6] (Figure 1). Regarding members of the CIN group, AtTCP2, AtTCP3, AtTCP4, AtTCP10, and AtTCP24 are post-transcriptionally regulated by the microRNA miR319, while AtTCP5, AtTCP13, and AtTCP17 are not [7] (Figure 1).

TCP proteins form dimers in solution which are essential for DNA binding [9,10]. DNA-binding preference studies indicated that the consensus binding sequence of class I TCPs is GTGGGNCC, while class II TCPs show a preference for the sequence GTGGNCCC [9,11,12]. These preferences are determined by one residue (Gly in class I or Asp in class II) located in the N-terminal basic region of the TCP domain [13]. Structural studies revealed that the TCP domain adopts a non-canonical basic-helix-loop-helix structure with three consecutive short β-strands followed by a helix-loop-helix motif [14]. More recently, crystal structures of DNA complexes of both classes of TCP domains revealed that TCPs are a unique and novel transcription factor family with a distinct DNA recognition and binding mechanism. TCP domain homodimers adopt a three-site recognition mode of DNA, mainly through a short pair of β-strands formed in the dimer interface and two basic flexible loops from the N-terminus of each TCP domain monomer [15]. This mechanism of DNA binding explains the manner in which a single residue determines the binding preferences of the two TCP classes. In addition, the TCP domain displays broad specificity for DNA sequences even shorter than the consensus [15], adding further complexity to the regulatory network of plant TCP transcription factors.

TCP proteins participate in the regulation of numerous processes of growth and development during the life cycle of plants, such as germination, photomorphogenesis, thermomorphogenesis, leaf development, flowering, development of floral organs, outgrowth of shoot branches, pollen development, circadian rhythms, cell cycle regulation, defense against pathogens, and senescence, acting through the recruitment of other factors and the modulation of different hormonal pathways [16,17,18,19,20]. Since their discovery more than 20 years ago, a considerable advance has been made in the knowledge of the roles of TCP transcription factors in plant reproductive development in diverse species. In this review, we specifically address the biological functions of TCPs in processes related to plant reproduction and the mechanisms by which they modulate flowering and the growth and development of floral organs and reproductive structures. A picture emerges in which the diversification of the TCP family was accompanied by the acquisition of multiple functions and the recruitment of groups of TCPs to specific processes in certain plant lineages. We discuss current knowledge about the action of the TCPs and raise some questions for future research.

## 2. Class I and Class II TCPs Regulate Flowering Time Acting at Different Levels

The floral transition is a critical developmental phase for reproductive success and is tightly controlled by a complex genetic network in response to various environmental (photoperiod, vernalization, and temperature) and developmental (gibberellin, aging, and the autonomous) pathways. These flowering signals ultimately converge on the regulation of floral integrators, such as the mobile florigen FLOWERING LOCUS T (FT) and the MADS-box transcription factor SUPPRESSOR OF OVEREXPRESSION OF CONSTANS1 (SOC1), which in turn activate downstream floral-meristem-identity genes, including *LEAFY* (*LFY*) and *APETALA1* (*AP1*), to initiate the generation of floral meristems and organ primordia [21,22]. The convergence of different pathways on a common set of genes may enable the integration of different responses, so that the plant can produce a coordinated flowering response under conditions in which multiple environmental parameters are changing simultaneously.

**Figure 2:**
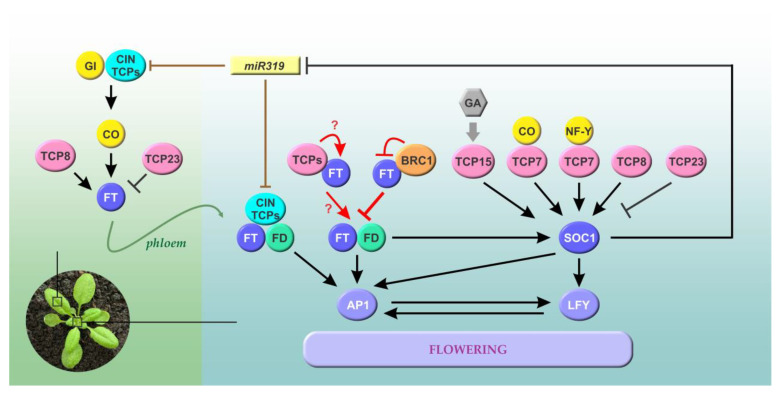
The roles of members of the TCP family in the regulation of flowering time in Arabidopsis. Class I and CIN TCPs directly or indirectly regulate the expression of *FT*, most likely in leaves (left, region shaded in green). Several TCPs also interact with FT or the FT-FD module to affect the expression of floral-meristem-identity genes, such as *AP1* and *LFY*, in the meristem. Class I TCPs directly modulate the expression of *SOC1*, another flowering time integrator. GA induces *SOC1* in part through the modulation of class I TCPs. Since SOC1 represses genes encoding miR319, a positive feedback loop is established, which connects class I and class II TCPs acting at multiple levels. Class I and CIN TCPs are depicted in pink and cyan, respectively. BRC1 (CYC/TB1 clade) is shown in orange. Transcriptional regulations are shown in black, while effects on protein activity are shown in red. Brown lines denote post-transcriptional regulation of CIN TCPs by miR319, and the green line shows the movement of FT from leaves to the apical meristem through the phloem. Arrows and T-shaped lines denote positive and negative regulation, respectively. See text for details.

In Arabidopsis, TCP transcription factors participate in the regulation of photoperiodic flowering. Although their molecular mechanisms of action have not been fully elucidated, TCPs from both classes were found to adopt different roles during the floral transition through interaction with other transcriptional regulators and cofactors. Moreover, a hierarchical interaction of TCPs from both classes was observed. Genetic studies in mutant and overexpressing plants evidenced that CIN TCPs and a subgroup of related class I TCPs, integrated by AtTCP7, AtTCP8, AtTCP14, and AtTCP15, function as positive regulators of flowering, whereas BRC1/AtTCP18 (CYC/TB1 clade) and class I AtTCP20, AtTCP22, and AtTCP23 act as flowering repressors [7,11,23,24,25,26,27,28,29,30,31,32] (Figure 2), revealing an intricate scenario for the function of TCPs in the control of floral transition. The miR319-targeted CIN TCPs, including AtTCP2, AtTCP3, AtTCP4, AtTCP10, and AtTCP24, act as positive regulators of the Arabidopsis photoperiodic flowering pathway by direct induction of the main photoperiod-responsive regulator *CONSTANS* (*CO*), an activator of *FT* transcription [27,28]. This subgroup of CIN TCPs interacts with the flowering activators FLOWERING BHLH (FBHs) and PHYTOCROME AND FLOWERING TIME1 (PFT1) to activate *CO* transcription [27,28]. In addition, the interaction of AtTCP4 with the flowering activator GIGANTEA (GI) enhances its DNA-binding ability onto the *CO* promoter, facilitating *CO* transcription [27].

Once synthesized in leaves, FT travels to the shoot apex where it interacts with FD to induce floral-meristem-identity genes [33]. Interrelations of TCPs with the FT-FD module, a key component of photoperiodic flowering, occur at different levels. The class I AtTCP8 activates the expression of *FT* to induce flowering [32], whereas the CIN TCPs not regulated by miR319, AtTCP5, AtTCP13, and AtTCP17 incorporate into the FT-FD module by facilitating the accessibility of FD to the *AP1* promoter [30]. The miR319-regulated CIN TCPs also interact with FD, and AtTCP4 directly regulates *AP1* expression. As a consequence, the floral-meristem-identity genes *LFY* and *FUL* are positively regulated by CIN TCPs [30]. In addition, TCPs from both classes (including positive and negative regulators of flowering) have been reported as interacting partners of FT [25,30,34,35] and proposed as differential mediators of the action of FT and TFL1, two closely related transcription cofactors with antagonist roles in flowering [34]. Different to FD, most class I TCPs interact with FT and a TFL1 mutant that mimics FT, but the interaction with TFL1 is significantly weaker [34], and BRC1 interacts specifically with FT, but not with TFL1 [25]. In the case of BRC1, it was observed that interaction with FT inhibits its function. As a result, and antagonistically to members of the CIN clade, BRC1 represses the expression of the FT downstream genes *AP1*, *LFY*, and *FUL* [25,30]. Since BRC1 is expressed in axillary buds, it delays floral transition specifically in axillary meristems [25]. The interaction between FT and FD is mediated by 14-3-3 proteins, but these are not necessary for the formation of the FT–BRC1 complex, which requires a different region of FT. Thus, BRC1 could bind to the FT–14-3-3–FD complex and inhibit its transcriptional activation function [25]. It will be interesting to find out the mechanisms by which other TCPs modulate FT activity through protein–protein interactions and the effect of these interactions on the flowering pathways. It can be speculated that the relative levels of TCPs acting as positive and negative flowering regulators would determine the effect on the activity of FT, and on flowering time, through the formation of different FT–TCP complexes.

Class I AtTCP7, AtTCP8, and AtTCP15 positively regulate Arabidopsis flowering mainly through direct regulation of the expression of the flowering integrator *SOC1* [29,31,32]. In turn, *SOC1* downregulates the expression of *miR319* genes, thus increasing class II AtTCP3 and AtTCP4 levels [28], possibly to accelerate reproductive development. Li et al. [31] found that the transcriptional activity of AtTCP7 on the *SOC1* promoter is enhanced by the interaction with the flowering time regulators NF-Ys and CO, while AtTCP15 was found to act as a mediator of the response of *SOC1* to gibberellin [29]. Meanwhile, AtTCP23 represses floral transition by affecting *FT* and *SOC1* expression [32]. The evidence suggests that AtTCP23 would repress *SOC1* expression by inhibiting the DNA binding or transcriptional activity of an activator of *SOC1* or facilitating the activity of a *SOC1* repressor [32]. Two other class I AtTCPs, AtTCP20 and AtTCP22, were found to delay flowering acting on the circadian clock, by regulating the expression of the clock gene *CIRCADIAN CLOCK ASSOCIATED1* (*CCA1*) [26]. 

Although TCP family proteins perform multiple functions in the regulation of flowering, the underlying molecular bases of their functional specificity are almost unknown and further studies are required. Since class II TCPs are divided into two clades with specific functions in plant development, the opposite roles of CIN TCPs and BRC1 in flowering could be attributed to structural properties or protein domains specific to each clade. For class I TCPs, subdivisions are less clear and redundant roles for several members in various processes were reported [16,36]. A recent report indicating that the N-terminal region located upstream of the TCP domain is responsible for the antagonistic effect of AtTCP8 and AtTCP23 on flowering seems to shed light on this aspect [32]. The situation becomes more complex since it was observed that TCPs can act as either activators or repressors, depending on the species. The miR319-targeted CIN TCPs promote flowering in *Arabidopsis*, but suppress it in tomatoes (*Solanum lycopersicum*) [37,38]. The miR319-targeted LANCEOLATE (LA/SlTCP4) represses floral transition by increasing gibberellin concentrations and inactivating the expression of *SFT* (*FT*) in the leaves and *AP1* at the shoot apex [38]. Therefore, the specific roles of TCP members in the regulation of flowering need to be evaluated in other species, especially those that do not have the photoperiodic pathway.

In summary, TCP family proteins participate in the flowering pathways by acting at different levels and by modulating the expression of both flowering time-related and floral-meristem-identity genes (Figure 2). Different members of both classes can act as floral activators or repressors in Arabidopsis, and this seems not to be conserved for the related members in other species. These functional differences could be due to the interaction of TCPs with different partners or the regulation of different target genes, which would lead to a different regulation of the activity and/or expression of other flowering regulators. Thereby, the modulation of the expression and activity of TCPs by environmental conditions and hormonal pathways would converge to adjust the timing of flowering with the growth conditions, ensuring reproductive success.

## 3. CYCLOIDEA-like TCPs and the Determination of Flower Symmetry

### 3.1. Molecular Mechanisms Involved in the Determination of Flower Symmetry in Antirrhinum majus

The importance of TCP transcription factors for the regulation of flower development was evident even before the family was described. In fact, the “C” in TCP refers to one of the founding members of the family, CYCLOIDEA (CYC) from *Antirrhinum majus* (snapdragon) [1]. The name refers to a mutant with “rounded”, radially symmetrical flowers, instead of the characteristic zygomorphic (bilaterally symmetrical) flowers of the species, with one small abaxial (ventral), two large adaxial (dorsal), and two lateral petals, resembling the face of a dragon [39]. Asymmetry is also present in whorl three, which contains two ventral and two lateral stamens, together with one arrested dorsal stamen or staminode. In flowers of the *cyc* mutant, the dorsal staminode develops into a fertile stamen and lateral petals become ventralized. Additional mutation of a related *TCP* gene, *DICHOTOMA* (*DICH*), causes full ventralization of dorsal and lateral petals and the development of extra petals and two fertile stamens in the dorsal region [40]. *AmCYC* and *AmDICH* are expressed in the adaxial domain of the floral meristem and their expression persists in adaxial organs until the late stages of flower development, indicating that they are required to suppress organ development and ventralization. In addition, ventralization of the lateral petals observed in *cyc* and *cyc dich* mutants suggests a cell non-autonomous effect of these transcription factors.

**Figure 3:**
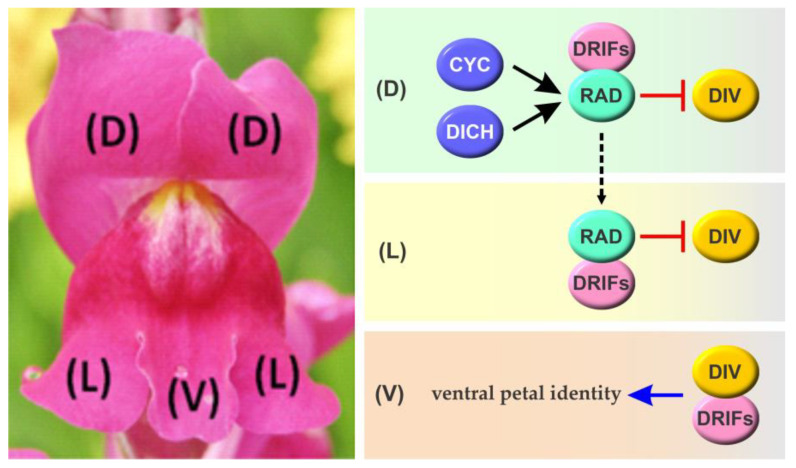
Scheme of the molecular interactions involved in the development of corolla zygomorphy in *A. majus*. The TCP transcription factors AmCYC and AmDICH induce the expression of RAD in dorsal petals (D), leading to the sequestration of DRIFs through protein–protein interactions and thus preventing DIV activity and ventralization. Similarly, the movement of RAD or its mRNA would prevent the ventralization of lateral petals (L). In the absence of RAD, DIV interacts with DRIFs leading to the activation of genetic programs related to ventral petal identity specification. Transcriptional regulations are shown in black, while effects on protein activity are shown in red. The broken line denotes the possible movement of RAD or its transcript from distal to lateral petals. Arrows and T-shaped lines denote positive and negative regulation, respectively. See text for details.

Mechanistically, AmCYC and AmDICH induce the expression of *RADIALIS* (*RAD*), which encodes a MYB transcription factor of the SANT/MYB subfamily, through direct transcriptional regulation [41,42] (Figure 3). Induction of *RAD* in dorsal petals inhibits the activity of another *MYB* gene, *DIVARICATA* (*DIV*). Notably, *DIV* is expressed throughout the flower, suggesting that RAD exerts a post-transcriptional effect on DIV activity, possibly through protein–protein interactions [43]. This is achieved by competition for binding to DIVARICATA AND RADIALIS INTERACTING FACTORs (DRIFs) 1 and 2, required for the activating function of DIV on its downstream targets [44]. In the absence of RAD, as in ventral petals in the wild type and all petals in *cyc dich* mutants, DIV–DRIF complexes form, thus conferring ventral identity (Figure 3). In the presence of RAD, RAD–DRIF complexes form, thus inhibiting DIV activity and suppressing ventral identity. Prevention of ventralization of lateral petals in the wild type, where RAD is not expressed, would be achieved by interorgan trafficking of RAD from the dorsal region [41] (Figure 3). Notably, a similar system involving three MYB proteins was shown to be involved in regulating cell expansion in the pericarp during tomato fruit development [45]. This led to the proposal that the regulatory module involving RAD, DIV, and DRIFs was adopted for the establishment of flower symmetry from a pre-existing module involved in carpel development [46]. This is also supported by the fact that *RAD*, *DIV*, and *DRIFs* are expressed in ovaries and developing fruits in *A. majus*.

### 3.2. CYC-like TCPs in Other Eudicots

AmCYC and AmDICH belong to the ECE clade, also named CYC/TB1, of class II TCP transcription factors, characterized by the presence of a conserved R-domain and an ECE (glutamate-cysteine-glutamate)-motif outside the TCP domain [4] (Figure 1). While members of the CIN clade of class II TCPs are present in all Embryophytes, those of the ECE clade are restricted to angiosperms [3]. Particularly, *AmCYC* and *AmDICH* belong to the *CYC2* group that evolved from an ancestral *ECE CYC/TB1* gene after two sequential duplications within core eudicots [4,47]. It was proposed that *AmCYC* and *AmDICH* originated after a whole-genome duplication event that occurred within the Plantaginaceae about 50 million years ago, which is supported by the presence of these genes in syntenic regions that contain dozens of homologous gene pairs [48]. Notably, *CYC2 TCP* genes are present not only in species with zygomorphic flowers, but also in those with radially symmetrical flowers [47]. In *Arabidopsis thaliana*, for example, the single *CYC2 TCP* gene *AtTCP1* does not affect flower symmetry and has been implicated in the regulation of leaf development and hormonal responses [49,50]. Notably, just like *AmCYC* and *AmDICH*, *AtTCP1* is expressed early in adaxial regions of the Arabidopsis flower meristem but expression ceases at later stages, thus precluding an effect on organ growth [51]. In another Brassicaceae, *Iberis amara*, adaxial *IaTCP1* expression persists at later stages of petal development and this is correlated with growth arrest of the dorsal petals and the development of flowers with bilateral symmetry [52]. This led to the proposal that a pre-existing state of asymmetric expression of *CYC2 TCP* genes may be a substrate for the evolution of zygomorphy in different plant lineages [53]. In fact, current evidence indicates that zygomorphy evolved independently multiple times within core eudicots [54] and that this is frequently related to changes in the function or expression of *CYC2 TCP* genes [55]. In addition, several documented cases of reversion from zygomorphy to radial symmetry also involved changes that disrupted the asymmetric expression or abolished the function of *CYC2 TCP* genes [56,57,58,59].

Changes in floral symmetry were probably fixed since they may confer advantages for plant reproduction and pollination efficiency [60,61,62]. In this sense, studies in the Brassicaceae *Erysimum mediohispanicum* showed that plants with zygomorphic flowers received more visits from pollinators and produced a larger number of seeds than plants with radially symmetrical flowers [60]. In *A. majus* and other Plantaginaceae, the showy flowers with a large dorsal petal are specialized for bee pollination. Meanwhile, the loss of a *CYC2* gene paralog, together with the corresponding *RAD* and *DIV* orthologs, led to the loss of zygomorphy associated with a shift to wind pollination in the Plantaginaceae *Plantago major* [57]. Thus, *CYC2 TCP* genes can be considered as major players in the determination of plant reproductive strategies through changes in flower morphology.

At the cellular level, CYC2 TCP proteins establish organ cell identity and regulate organ size by affecting either cell proliferation or cell expansion. *A. majus* CYC, for example, promotes cell expansion in dorsal petals and inhibits cell proliferation in dorsal stamens by regulating the expression of the *CYCLIN D3B* gene [42,63], while *I. amara* TCP1 restricts dorsal petal growth through negative regulation of cell proliferation [52]. Notably, the positive effect of *A. majus* CYC and the negative effect of *I. amara* TCP1 on petal growth are conserved when these transcription factors are expressed in *A. thaliana* [41,52]. The molecular nature of these apparent divergent effects of closely related members of the TCP family is unknown but clearly indicates the participation of different regulatory circuits that most likely involve protein–protein as well as protein–DNA interactions. 

### 3.3. CYC-like TCPs and the Specification of Flower Identity in Asteraceae

In addition to participating in the establishment of flower form, *CYC2 TCP* genes are involved in the development of the different types of flowers formed in the inflorescence (capitulum) of Asteraceae [64]. The inflorescence of these plants is composed of a disk with multiple fertile, radially symmetrical flowers and rays formed by infertile, zygomorphic flowers. Analysis of *TCP* genes indicated that the *CYC2* clade is particularly enlarged in members of this family due to duplications that occurred within the Asteraceae (Figure 1) and that some of these genes were recruited for the determination of flower identity within the capitulum [65,66,67]. Particularly, *CYC2 TCP* genes are expressed in ray flower primordia and their loss of function causes the radialization of ray flowers, which also develop reproductive organs in some cases [68,69,70]. This is observed in the *tubular ray flower* (*turf*) mutant of sunflower (*Helianthus annuus*) in which a transposable element was inserted within the coding region of the *HaCYC2c* gene [68,69]. Interestingly, a transposon insertion in the promoter region of the same gene leads to its ectopic expression throughout the capitulum and the development of ray-like flowers in the disk, thus showing the role of *HaCYC2c* in determining ray flower identity [69]. In *Gerbera hybrida*, three *CYC2 TCP* genes, *GhCYC2*, *GhCYC3*, and *GhCYC4*, are involved in the determination of ray flower identity and also participate in the regulation of ray petal growth through changes in cell proliferation [65,71]. Another *G. hybrida CYC2 TCP* gene, *GhCYC5*, affects flower density in the capitulum, suggesting that it may be involved in the promotion of flower initiation [71]. Recent evidence indicated that two CIN TCP proteins are also involved in the development of ray flower primordia, acting upstream of *CYC2 TCP* genes [72].

### 3.4. CYC-like TCPs and the Specification of Other Flower Characteristics Associated with Symmetry

In particular cases, other flower characteristics, in addition to floral symmetry, are under the control of CYC2 TCP proteins. Studies in the *peloric* variety of gloxinia (*Sinningia speciosa*) indicated that a 10 bp deletion in the coding sequence of the *CYC2* clade gene *SsCYC* caused a change from horizontal to upright orientation of the flower, in addition to radialization [59]. The horizontal orientation of the flower in zygomorphic varieties is caused by asymmetric growth at the base of the corolla, which is related to patterns of *SsCYC* expression. In another example, flower coloration is strongly associated with floral symmetry in species of the group *Chrysanthemum* s.l., which present yellow disk flowers and either colored or white ray flowers. The association of flower color and floral symmetry relies on the fact that a carotenoid cleavage dioxygenase gene (*CCD4a*) fell under the control of CYC2g and is expressed exclusively in ray flowers [73]. This leads to the degradation of carotenoid pigments in ray flowers and the production of white petals, while species with a dysfunctional *CYC2g* gene show reduced zygomorphy and yellow pigmentation of ray flowers [73]. Association of pigmentation with floral symmetry has also been observed at the level of individual flowers in *Torenia fournieri*, in which petals show differences in pigmentation due to changes in anthocyanin production. It was observed that silencing of the *TCP* gene *TfCYC2* causes flower radialization and the development of dorsal petals with a strong violet coloration, while its overexpression reduces pigment accumulation in all petals [74]. These effects are due to the direct repression of a gene encoding a MYB transcription factor involved in anthocyanin production by TfCYC2.

### 3.5. CYC/TB1 TCPs and the Specification of Flower Characteristics in Basal Eudicots and Monocots

CYC/TB1 TCP proteins are also involved in flower development in basal eudicots and in monocots. *CYC-like* (*CYL*) genes in *Eschscholzia californica* and *Cysticapnos vesicaria*, two basal eudicots with radially symmetrical and zygomorphic flowers, respectively, were shown to be involved in the promotion of stamen initiation and growth and the regulation of petal size [75]. This is opposite to observations for *CYC2 TCP* genes in core eudicots, where they promote stamen developmental arrest. In addition, silencing of *C. vesicaria CYL* genes causes the homeotic transformation of sepals into petals related to the induction of B-class *MADS-box* genes [75]. Partial loss of bilateral symmetry was also observed after silencing *C. vesicaria CYL* genes, suggesting an involvement in the determination of the floral symmetry of these genes as well. In rice (*Oryza sativa*), the *CYC/TB1* gene *RETARDED PALEA1* (*REP1*) is involved in the determination of the identity of the palea, a protective bract- or sepal-like structure that develops on one side of the flower, opposite to another protective structure termed the lemma. In *rep1* mutants, palea development is retarded and the modified palea shows characteristics of the wild-type lemma [76]. Notably, *REP1* is asymmetrically expressed in the palea primordium and its ectopic expression disrupts palea bilateral symmetry, indicating that *CYC/TB1* genes were also recruited to modify floral symmetry during monocot evolution. However, the involvement of *CYC/TB1* genes in the evolution of flower zygomorphy in monocots seems to be less frequent, even if this trait developed independently several times within this lineage [77].

## 4. CIN TCPs and the Regulation of Petal Differentiation and Growth

In addition to the role of CYC/TB1 clade class II TCPs in the control of flower symmetry, several reports indicate that members of the CIN clade also affect petal growth and shape, although not in an asymmetric manner. The first characterized member of the CIN clade, *A. majus* CINCINNATA (CIN), affects epidermal cell differentiation and the growth of petal lobes [78]. *CIN* loss-of-function mutants contain flat epidermal cells instead of the conical cells observed in the wild type. The effect of CIN on petal growth is probably related to the regulation of cell proliferation, as also observed in leaves. However, while CIN promotes the growth of petals, it causes growth arrest in leaves. The reason for this opposite behavior is unknown.

**Figure 4:**
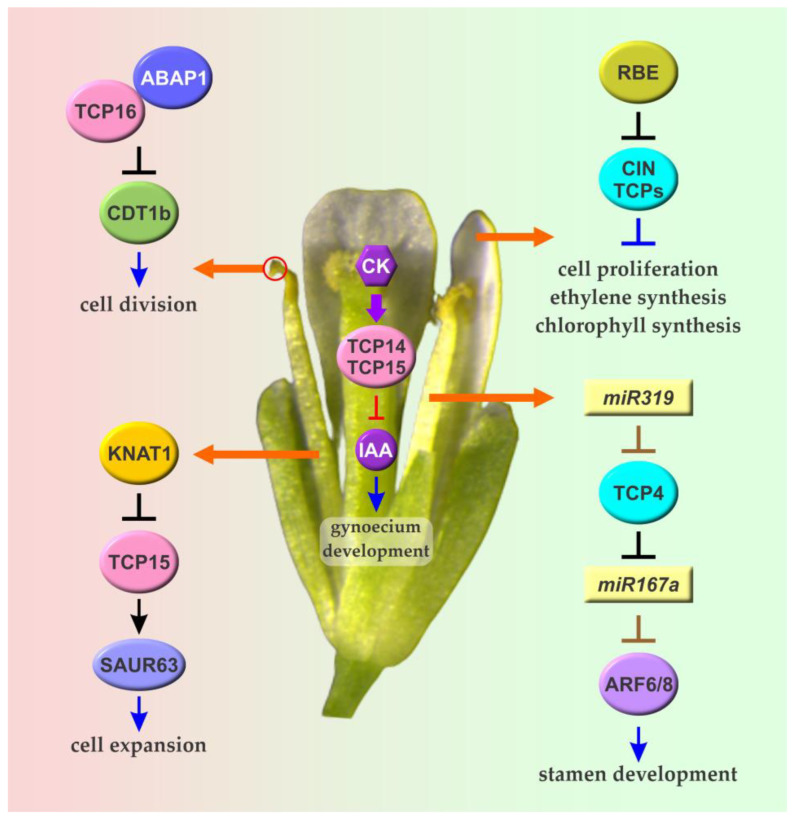
The role of Arabidopsis TCPs in the development of flower organs. CIN TCPs repress cell proliferation and promote cell differentiation in petals. Repression of CIN TCP expression by RBE at the early stages of petal development is required for optimal petal growth. AtTCP4 and other CIN TCPs regulated by miR319 also participate in the modulation of hormonal responses related to stamen maturation influencing the expression of miR167-dependent AUXIN RESPONSE FACTORs (ARFs) 6 and 8. AtTCP15 and related class I TCPs promote cell expansion in stamen filaments through direct regulation of a group of *SAUR* genes. These TCPs also participate in gynoecium development acting on cytokinin- and auxin-dependent processes. Another class I protein, AtTCP16, affects cell division in the male gametophyte and is required for optimal pollen development. Class I and CIN TCPs are depicted in pink and cyan, respectively. Transcriptional regulations are shown in black. Brown lines denote post-transcriptional regulation by miRNAs. Arrows and T-shaped lines denote positive and negative regulation, respectively. Details about these and other roles of TCPs during the development of flower organs are provided in Section 4 and Section 5.

The role of CIN TCPs in the differentiation of petal cells seems to be conserved in Petunia, where silencing of the *CIN TCP* gene *PhLA* also produces petals with flattened epidermal cells [79]. However, silencing of *PhLA* also causes cell enlargement and increased petal curvature due to increased growth of the distal portion of the petals, contrary to observations in Antirrhinum *cin* mutants. In Arabidopsis, joint loss of function of several *CIN TCP* genes, namely *AtTCP3*, *AtTCP4*, *AtTCP5*, and *AtTCP10*, causes the development of wavy and serrated petals, similar to the effect observed in leaves [80]. Similar observations were made after the expression of dominant repressor forms (TCP-SRDX) of AtTCP3 and AtTCP5 [80,81], and this strategy was used to produce changes in the morphology of flowers with ornamental value [82,83]. Another study indicated that silencing the closely related Arabidopsis *CIN TCP* genes *AtTCP5*, *AtTCP13*, and *AtTCP17* causes an increase in the width of the petal claw and blade, while overexpression of *AtTCP5* causes opposite changes [84]. AtTCP5 and related TCPs affect petal growth through inhibition of cell proliferation, affecting the number and duration of mitotic events within the organ. At the early stages of petal development, expression of the TCPs is repressed by the C2H2 zinc finger transcription factor RABBIT EARS (RBE), thus allowing cell proliferation and organ growth [84] (Figure 4). This repression is relieved at later stages, causing the cessation of cell proliferation and the transition to a cell expansion phase. In addition to cell proliferation, it was proposed that AtTCP5 and related TCPs affect ethylene biosynthesis through the repression of *ACC SYNTHASE 2* (*ACS2*) and *ACC OXIDASE 2* (*ACO2*) [85]. In agreement with this, treatment with an ethylene synthesis inhibitor rescues the defects of a mutant in *AtTCP5* and related genes. RBE also represses the expression of the *CIN TCP* gene *AtTCP4*, which belongs to the group of miR319-regulated TCPs [86], suggesting that multiple members of the CIN clade perform similar functions during petal development. This agrees with observations indicating that mutants in *MIR319a* exhibit narrow and short petals [87].

Changes in petal characteristics may be important for plant reproduction, mainly in species that require the action of pollinators. Conical epidermal cells in petals alter light absorption, influencing petal brightness, color intensity, and petal temperature, which is related to the production of volatile compounds, thus favoring pollinator attraction through visual and olfactory cues [88,89]. In addition, conical cells provide a better grip for pollinator landing [90]. Thus, changes in the function of CIN TCPs may influence the reproductive strategies of plants through modification of petal cell characteristics.

In addition to affecting petal growth, the activity of CIN TCPs is required to prevent petal greening in Arabidopsis. In wild-type plants, plastids located at the base of petals retain chlorophyll, thus conferring a green color to this region of the organ, which is otherwise white. In plants with defective CIN TCP activity, the green region is extended to the whole petal due to a lack of inhibition of chlorophyll biosynthesis in distal parts of the petal [91]. It was found that some genes related to chlorophyll biosynthesis are directly repressed by AtTCP4 and probably other CIN TCPs [91]. Whether this activity of CIN TCPs is also relevant for other processes that imply organ degreening, such as leaf senescence or fruit maturation, is unknown.

It can be concluded that multiple TCPs from the CIN clade are involved in determining petal characteristics, mainly acting on processes related to cell proliferation and differentiation. This is reminiscent of the known role of CIN TCPs during leaf development, where they act as maturation factors that limit cell proliferation and promote cell differentiation [92]. To what extent the effects of the TCPs on leaf and petal development are due to their action on overlapping molecular pathways is unknown.

## 5. TCPs and the Modulation of Reproductive Organ Development and Growth

### 5.1. TCPs and the Regulation of Stamen Growth and Development

The action of class I TCPs has been linked to the growth and development of reproductive floral organs. The Arabidopsis gene encoding the class I TCP AtTCP15 is expressed in stamen filaments at pre-anthesis stages and is involved in promoting stamen elongation, mainly acting on the activation of a group of genes from the *SMALL AUXIN UP RNA* (*SAUR*) family through direct binding to their promoters [93] (Figure 4). The products of *SAUR* genes promote cell expansion by affecting the activity of plasma membrane H^+^-ATPases in response to different hormones [94], and it was shown that AtTCP15 and related class I TCPs are involved in mediating the effect of gibberellins on stamen growth [93]. Expression of *AtTCP15* is directly repressed by the homeodomain protein BREVIPEDICELLUS (BP), also named KNAT1, and possibly by other related homeodomain transcription factors, at late stages of stamen development, presumably to slow down stamen growth once it reaches the top of the pistil [95] (Figure 4). Expression of a repressor form of AtTCP15 produces flowers with short stamens, while overexpression of the native protein causes the opposite effect. In both cases, however, seed production seems to be affected, revealing the importance of correct stamen filament elongation for efficient reproduction in autogamous plants such as *A. thaliana*.

A divergent class I TCP, AtTCP16, was related to the early stages of Arabidopsis pollen development. *AtTCP16* is expressed in developing microspores and its silencing produces abnormal pollen development already observable at the unicellular stage [96]. As a consequence, pollen grains from *AtTCP16* defective plants show an abnormal structure and are not viable. It was reported that AtTCP16 binds to the promoter of the DNA replication factor gene *CDT1b* in a complex with the Armadillo BTB Arabidopsis protein 1 (ABAP1) to repress its expression and regulate the first asymmetric mitotic division in the male gametophyte [97] (Figure 4). In addition, AtTCP16 represses the expression of the intracellular copper transporter COPT3 and changes in *AtTCP16* function alter the growth of plants under varying copper concentrations [98]. Since *COPT3* is highly expressed in pollen and *copt3* mutants show defective pollen under copper deficiency conditions, it was postulated that AtTCP16 may affect pollen development also by influencing copper homeostasis [98]. TCP16-like proteins arose in rosids and have a distinct DNA-binding specificity in relation to other class I TCPs [13], suggesting that they were probably recruited for the modulation of pollen development during the evolution of this lineage of plants. 

Class II TCPs may also be involved in stamen development, although a detailed analysis of their mode of action is still lacking. As mentioned in Section 3, CYC TCPs from different species affect stamen development in addition to petal growth. In addition, a mutant in *MIR319a* shows defective anther development and the expression of a miR319-resistant form of AtTCP4 abolishes stamen growth [87]. It was proposed that miR319-targeted CIN TCPs coordinate hormonal responses related to stamen development and maturation acting on the activity of homeodomain and auxin response factors [99] (Figure 4). In addition, it was reported that the miR319-targeted CIN TCP AtTCP24 affects secondary cell wall thickening in the anther endothecium and its overexpression leads to decreased anther dehiscence and sterility [100].

### 5.2. TCPs and the Regulation of Gynoecium Development

AtTCP15 and related class I TCPs also participate in gynoecium development through the modulation of the cross-talk between auxins and cytokinins [101] (Figure 4). *AtTCP15* is preferentially expressed in valves and in the replum, and disruption of *AtTCP15* function causes altered responses to cytokinins, presumably due to changes in auxin levels. Particularly, cytokinin treatment of loss-of-function mutants in *AtTCP15* and a related *TCP* gene causes replum enlargement and the development of stigma-like structures along the replum, while treatment of plants that overexpress *AtTCP15* suppresses replum formation and promotes the development of undifferentiated structures in apical and medial parts of the gynoecium [101]. It was proposed that the correct development of the different tissues that compose the gynoecium requires a correct balance of auxin levels and cytokinin responses and that AtTCP15 and other class I TCPs are involved in establishing this balance [101]. Interestingly, BP/KNAT1, which acts as a repressor of *AtTCP15* during stamen elongation [95], also affects replum and valve size [102], raising the possibility that this repression also has a role during gynoecium development. In addition, AtTCP15 was shown to interact with several transcription factors related to different aspects of gynoecium development, suggesting that it may act as a hub of the molecular network involved in this process [103]. This study also suggested the participation of additional TCPs from both classes in the process, which deserves further investigation. In this sense, it was recently reported that in Arabidopsis *jaw-D* plants, in which the expression of several CIN TCPs is reduced as a consequence of miR319 overexpression, the silique replum is enlarged relative to that of wild-type plants, and the same happens when a dominant repressor form of the CIN protein AtTCP3 (AtTCP3-SRDX) is expressed from its own gene promoter [104]. Contrary to that, increased expression of *AtTCP3* or its putative *Brassica napus* ortholog in the respective species reduces replum width and lignification, conferring increased resistance to shattering [104]. In addition, CIN TCPs were related to ovule development since overexpression of either *AtTCP3* or *AtTCP5* causes ovule abortion, and loss of function of multiple *CIN TCP* genes disrupts ovule initiation and arrangement [105]. The function of CIN TCPs is modulated by interaction with the transcriptional repressor SPOROCYTELESS/NOZZLE (SPL/NZZ), which in turn recruits TOPLESS co-repressors. It was proposed that SPL/NZZ acts as an adaptor to recruit TOPLESS to TCP target genes, thus preventing excessive expression of these genes which would disrupt ovule development [105]. Hence, correct regulation of the activity of CIN TCPs is essential for ovule development, but the mechanism of action of these TCPs in this process remains unknown. 

In summary, the role of TCPs in the development of reproductive structures deserves further investigation and is highly likely that different members of the family have multiple roles during this process. In addition, much is yet to be learned about the molecular interactions related to their participation in reproductive development. 

## 6. TB1-like TCPs and the Regulation of Inflorescence Architecture in Grasses

Like *A. majus* CYC, maize (*Zea mays*) TB1 is another founding member of the family, responsible for the “T” in TCP [1]. It was recognized as a determinant of apical dominance through the inhibition of the growth of shoot axillary branches [106] and, like AmCYC, belongs to the ECE (or CYC/TB1) clade of class II TCPs. The function of TB1-like TCPs in the regulation of shoot branching is conserved among angiosperms [107,108,109,110,111,112], even if diversification of the CYC/TB1 clade seems to have occurred independently in monocot and dicot plants [4,66,113]. Several studies indicated that genes from the CYC/TB1 clade participate in the determination of inflorescence architecture in grasses (Poaceae). This function probably evolved after two rounds of duplications that took place early in this lineage, resulting in three different clades, named 18–20 by Mondragón-Palomino and Trontin [113]. The inflorescence of grasses shows a remarkable complexity and diversity and is generally formed by a central axis bearing branches and specialized structures called spikelets that contain the flowers [114]. Variations in the number and determinacy of branches and spikelets determine inflorescence architecture, as well as grain production and other important agronomic traits.

A *CYC/TB1* gene involved in the control of inflorescence architecture in maize is *BRANCH ANGLE DEFECTIVE 1* (*BAD1*), also named *WAVY AURICLE IN BLADE1* (*WAB1*) [115,116]. Unlike *TB1*, which belongs to group 18 of *CYC/TB1* genes from grasses, *BAD1/WAB1* belongs to group 20, together with *REP1*, involved in the control of rice floral zygomorphy and palea development as mentioned in Section 3.5 [76] (Figure 1). Specifically, *BAD1/WAB1* affects the angle of lateral branches that emerge from the main axis of the inflorescence controlling the growth of the pulvinus, a structure that separates the lateral branches from the main axis. Mutants in *BAD1/WAB1* show reduced pulvinus size, which results in a reduced angle of the lateral branches and a more compact inflorescence [115], together with a reduced number of inflorescence branches [116]. The gene is expressed in a region located between the inflorescence meristem and the branch meristem and may be involved in establishing the boundary between these structures probably acting on the expression of *LIGULELESS1* (*LG1*), a gene also involved in establishing leaf angle [116]. Notably, the loss of function of an ortholog of maize *BAD1/WAB1*, named *COMPOSITUM 1* (*COM1*) or *BRANCHED AND INDETERMINATE SPIKELET 1* (*BDI1*), causes an opposite effect on inflorescence branching in barley (*Hordeum vulgare*) [117,118]. In mutants of this gene, a lateral branch with an inflorescence-like structure develops instead of a spikelet, suggesting that it is involved in establishing meristem identity and/or determinacy. *COM1/BDI1* is expressed in similar regions as *BAD1/WAB1*, suggesting that its differential effect on inflorescence branching may be related to changes in the properties of the protein [117]. It was postulated that, instead of conferring boundary identity, *COM1/BDI1* may participate in signaling mechanisms that affect the activity of adjacent meristems. This specialized function of *COM1/BDI1* was probably relevant for the evolution of the typical unbranched inflorescence of barley and other Triticeae grasses.

Yet another *CYC/TB1* gene is involved in the development and growth of lateral spikelets in barley. Reduced function of this gene, known as *INTERMEDIUM-C* (*INT-C*) or *SIX-ROWED SPIKE 5* (*VRS5*), causes an increase in lateral spikelet fertility, leading to increased grain production [119]. The wild barley inflorescence contains two rows of grain-producing spikelets, while modern barley cultivars contain six. This is due to the presence of three fertile spikelets, one central and two lateral, at each node, while in two-rowed barley the development of the two lateral spikelets is inhibited [120]. Selection of specific *INT-C* alleles was probably involved in the development of six-rowed varieties during barley domestication [121]. The sorghum (*Sorghum bicolor*) CYC/TB1 transcription factor MULTISEEDED 1 (MSD1) is also involved in the control of spikelet fertility [122]. The sorghum inflorescence exhibits branches at the nodes, and each branch develops two types of terminal spikelets, sessile and pedicellate, with only the single sessile spikelet being fertile [123]. In mutants in *MSD1*, the two pedicellate spikelets are also fertile and produce grains. It was reported that MSD1 affects pedicellate spikelet development through positive regulation of jasmonic acid (JA) synthesis, which is supported by the fact that JA application rescues the phenotypic effects of the *msd1* mutation [122]. While *INT-C* is an ortholog of maize *TB1*, *MSD1* belongs to group 19 of *CYC/TB1* genes (Figure 1), indicating that genes from different groups were recruited to exert their function in spikelet fertility during the diversification of grasses.

*TB1* and its orthologs were also related to changes in inflorescence architecture in maize, rice, and wheat. In maize, TB1 affects the size and characteristics of the glume, a bract-like structure located at the base of the spikelet, and internode elongation in the ear [124]. This effect would be the consequence of the direct regulation of cell-cycle-related genes, as well as of *ETB1.2*, which encodes a YABBY transcription factor that promotes internode elongation, and *TGA1*, involved in glume elongation [124]. TB1 also influences the number of spikelets present in the cupules of the ear [125]. Alleles of *TB1* present in maize, which show increased *TB1* function or expression in relation to the ones present in teosinte, cause the development of more compact ears with a higher number of naked grains, thus improving harvest quality and grain production. In wheat (*Triticum aestivum*), changes in the activity of *TaTB1* were associated with the development of paired spikelets, which implies the development of two spikelets instead of a single one at given inflorescence nodes, thus resulting in increased grain production [126]. Allelic variation in *TaTB1* is associated with the development of paired spikelets in modern wheat varieties [126]. It was shown that wheat TB1 interacts with the flowering regulator FLOWERING LOCUS T1 and alters the inflorescence growth rate and the expression of meristem-identity genes. Interestingly, a similar interaction was reported for the respective Arabidopsis orthologs, BRC1 and FT [25] (see Section 2), suggesting the existence of conservation of a negative role of TB1 orthologs on flowering through FT. Dixon et al. [126] postulated that TB1 orthologs in cereals negatively control the rate of development of axillary meristems into spikelet meristems, thus influencing different aspects of inflorescence architecture and grain production.

## 7. Conclusions and Perspectives

In summary, TCP transcription factors from different clades exert multiple roles in the development of plant reproductive structures. This multiplicity of roles is probably the consequence of the diversification of the family that took place during land plant evolution, followed by neofunctionalization of the different members. It can be speculated that an ancestral function of TCPs in the regulation of cell proliferation and expansion was co-opted for the modulation of specific processes in different organs or structures through changes in expression patterns. This was probably accompanied by changes in protein characteristics that modified protein–DNA interactions and, perhaps more importantly, protein–protein interactions, thus integrating the TCPs into different molecular pathways. Particularly, several TCPs seem to exert their function by acting in close connection with specific hormonal pathways related to growth and differentiation. Thus, studying the molecular interactions used by the TCPs to exert their function would be essential to understand their mode of action. Considering the vast diversification observed in different plant lineages, this will require extensive studies in different species in addition to *A. thaliana*. Moreover, comparative studies of different members of the TCP family will be required to understand the molecular basis of their functional specificity. Eventually, affecting the function of specific TCPs could be a tool to modify aspects of plant reproductive structures that influence ornamental properties, reproductive efficiency, and seed production.

## Data Availability

Not applicable.
